# Teleconsultations for Eczema in CHildren (TECH) feasibility study: a mixed-methods study with adolescents and parents

**DOI:** 10.1093/skinhd/vzaf123

**Published:** 2026-01-30

**Authors:** Natalie King Stokes, Aoife Daly, Sarah McCusker, Manrup Hunjan, Ellen Vincent, Ashima Lowe, Carolyn Charman, Jonathan Mathers, Lea Solman, Susannah George, Esther Burden-Teh

**Affiliations:** Centre of Evidence Based Dermatology, School of Medicine, University of Nottingham, Nottingham, UK; Department of Dermatology, Guy’s and St Thomas’ NHS Foundation Trust, London, UK; Department of Dermatology, Belfast Health & Social Care Trust, Belfast, UK; Department of Dermatology, Royal Hospital for Children, NHS Greater Glasgow and Clyde, Glasgow, UK; Department of Dermatology, Chelsea and Westminster Hospital NHS Foundation Trust, London, UK; Patient co-Applicant; Department of Dermatology, Swansea Bay University Health Board, Swansea, UK; Department of Dermatology, Royal Devon University Healthcare NHS Foundation Trust, Exeter, UK; Applied Health Sciences, School of Health Sciences, College of Medicine and Health, University of Birmingham, Birmingham, UK; Department of Dermatology, Great Ormond Street Hospital for Children NHS Foundation Trust, London, UK; Department of Dermatology, Brighton General Hospital, University Hospitals Sussex NHS Foundation Trust, East Sussex, UK; Centre of Evidence Based Dermatology, School of Medicine, University of Nottingham, Nottingham, UK

## Abstract

**Background:**

Teleconsultations form a key component of the National Health Service’s Long Term Plan, outpatient recovery and ­transformation, and commitment to net zero. There is currently little evidence to inform their delivery within paediatric dermatology, a specialty that is increasingly difficult to access. Teleconsultations may offer convenience and widen access for patients, particularly for follow-up of chronic conditions such as eczema.

**Objectives:**

To explore patients’ and parents’ experiences and perceptions of teleconsultations for follow-up of eczema in secondary or tertiary care; to identify components of acceptability of different consultation types and explore how teleconsultations could be optimized; and to obtain views of a potential trial comparing face-to-face appointments with teleconsultations for paediatric eczema follow-up.

**Methods:**

A mixed-methods study comprising an online survey and qualitative interviews with patients and parents of children with eczema, who had experience of teleconsultations under secondary or tertiary care, was carried out. An online survey was completed by eligible adolescents and parents. The results informed subsequent semi-structured interviews, conducted with patients and parents, and responses were analysed thematically using framework analysis.

**Results:**

Survey responses from 31 parents and 20 adolescents were analysed. Overall, 41% were ‘satisfied or very satisfied’ following telephone appointments vs. 71% for video and 40% for online or mobile messaging. Preferred consultation type was face-to-face in 67%, with 31% preferring a combination of face-to-face and teleconsultations. Some 41% of participants were interested in participating in a trial comparing face-to-face and teleconsultations for eczema follow-up. Qualitative interviews were conducted with 10 parents and 2 adolescents. Six overarching themes were constructed from the interview data: the value of a dermatology appointment, what is important in an eczema consultation, concerns about patient assessment, complexity of acceptability, patient choice and optimizing teleconsultations.

**Conclusions:**

While there was a preference for face-to-face consultations, teleconsultations were acceptable in certain situations, such as for stable eczema or to avoid missing school. Patient perspectives of how teleconsultations could be improved include sending photographs before the appointment and clinician training. This study suggests that directly comparing face-to-face appointments with teleconsultations in a future trial is unlikely to be feasible. A hybrid intervention combining face-to-face and teleconsultation would be more acceptable to patients and parents.

## Teaser

This study explored the experiences and preferences of adolescents and parents having teleconsultations for follow-up of atopic eczema in secondary/tertiary care, through an online survey and qualitative interviews. Key findings included a preference for face-to-face consultations, but that teleconsultations were acceptable in certain situations. Participants wanted more choice and shared decision making to determine how they are followed up for eczema.

What is already known about this topic?

- Paediatric dermatology is increasingly difficult to access due to service closures and long waiting lists; eczema is the most common reason for referral to paediatric dermatology, often requiring multiple follow-ups.
- Teleconsultations for paediatric eczema follow-up may offer benefits such as widening access to appointments, reducing missed appointments, convenience, cost savings and sustainability.
- There is little evidence on the effectiveness and acceptability of teleconsultations for paediatric eczema compared with face-to-face appointments.

What does this study add?

- This study highlights the value and benefits of face-to-face appointments, particularly for initial assessments.
- Dissatisfaction with teleconsultations is predominantly due to lack of skin examination, lack of child involvement and communication difficulties.
- Most parents and adolescents prefer face-to-face appointments for follow-up of their eczema; others prefer a mix of face-to-face and teleconsultations; teleconsultations were acceptable in certain circumstances such as in stable eczema or to avoid missing school or work.

The National Health Service (NHS) Long Term Plan 2019 set out to redesign the traditional outpatient model, which was deemed outdated and unsustainable. Using technology to provide timely support to general practitioners (GPs), online booking systems, care closer to home and teleconsultations, it aimed to save 30 billion hospital visits and over £1 billion per year.^1^ The roll out of teleconsultations was expedited in response to the COVID-19 pandemic. A teleconsultation is a remote appointment given via telephone, video or asynchronously using digital platforms.^2^ Benefits of teleconsultations include convenience, widened access to healthcare, sustainability, and cost and time savings for patients and the NHS.^3^ Providing teleconsultations remains a key component of NHS outpatient recovery and transformation, which aims to provide better care at the right time and more choice over how patients access care.^4^

While teleconsultations have potential benefits, there are also disadvantages such as the inability to conduct a physical examination, technical challenges and digital exclusion of those without access to or the ability to use the technology.^3^ Paediatric teleconsultations provide additional challenges in communication, consent and child protection.^5^ Yet the provision of teleconsultations in paediatric dermatology could be beneficial, as this subspecialty is becoming increasingly difficult to access due to service closures and shortages of paediatric dermatologists.^6^ In particular, atopic eczema, the most common reason for referral to paediatric dermatology, could be a condition suitable for remote management, being a chronic condition often requiring multiple follow-ups, with self-management a key component of treatment.^7–9^

There is sparse evidence to inform whether teleconsultations can be implemented effectively for the follow-up of paediatric eczema. Four pre pandemic randomized controlled trials compared asynchronous online platforms with face-to-face follow-up of paediatric eczema.^10–13^ In these studies, the intervention comprised an online portal where parents submitted information about their child's eczema, including photographs, to which a specialist responded with treatment recommendations. Three studies showed patient-reported disease severity outcomes equivalent to face-to-face appointments.^10,12,13^ These studies are of limited relevance to UK practice, where telephone is the most common method of conducting a teleconsultation.^14^ In an interview study of eczema self-management in primary care, telephone consultations with GPs were viewed as less satisfactory by patients, compared with face-to-face appointments.^15^ The evidence for whether teleconsultations are acceptable to patients and parents for delivery of paediatric eczema follow-up in secondary care remains limited.

The aim of the study was to explore the experiences and preferences of adolescents and parents having teleconsultations for eczema in secondary or tertiary care, to inform the design of a clinical trial comparing teleconsultations with face-to-face appointments for paediatric eczema follow-up.

The study objectives were (i) to describe adolescents’ and parents’ experiences, views and preferences of teleconsultations and face-to-face consultations for eczema follow-up; (ii) to identify components of acceptability that are important to adolescents and parents when evaluating teleconsultations; (iii) to explore views on how teleconsultations could be delivered most effectively; and (iv) to explore views and willingness to be randomized in a potential trial comparing teleconsultations with face-to-face consultations for paediatric eczema follow-up.

## Patients and methods

This was a mixed-methods study comprising an online survey and qualitative semi-structured interviews. The study population was adolescents (aged 13–16 years) with atopic eczema under secondary or tertiary care dermatology and parents of children and adolescents with eczema. Patients were involved in the study design and implementation, including study protocol, interview and survey design, reviewing information leaflets and consent forms and survey piloting. We presented our study to the Centre for Evidence Based Dermatology (CEBD) patient panel, who advised on topics for our interview guide, based on their experiences of teleconsultations, and conduct of interviews. We had input throughout our study from our patient representative (E.V.).

### Survey

An online survey was advertised on social media [via the National Eczema Society, the British Skin Foundation, Eczema Outreach Support (EOS), the UK Dermatology Clinical Trials Network (UK DCTN) and CEBD charities, and paid Facebook adverts] and via EOS, UK DCTN and CEBD mailing lists in December 2022 for 12 weeks (Figure S1, Appendix S1; see Supporting Information). Adolescents (aged 13–16 years) and parents of children and adolescents (aged 0–16 years) who had had at least one follow-up teleconsultation (telephone, video or via mobile application) for eczema in secondary or tertiary care were eligible. Informed consent was taken at the start of the survey. A prize draw was offered on survey completion; however, after multiple fictitious responses were received, the prize was withdrawn and a protocol implemented to identify such responses (protocol amendment approved by the Research Ethics Committee). The survey was distributed via Jisc Online Survey, a secure, General Data Protection Regulation-compliant, encrypted platform. Data were exported directly into Microsoft Excel and anonymized. Fixed response categorical data were analysed with descriptive statistics and a simple content analysis undertaken with free-text data.

### Interviews

Interview participants were recruited via the online survey and further advertisement on social media and EOS, UK DCTN and CEBD mailing lists. Eligibility was rechecked and an age-appropriate study information sheet sent to interested individuals, with the opportunity to ask questions. Written consent was obtained via an electronic form (Microsoft Office 365). Limited interest in the interviews resulted in a convenience sample of 10 parents and 2 adolescents. Despite this, the experiences of participants of a range of ages and backgrounds were included, providing sufficient data for the purposes of our study (Table 1).

**Table 1:** Patient demographics (survey and interview)

Patient demographics	Survey participants: parents^a^ (*n* = 31)	Survey participants: adolescents (*n* = 20)	Survey participants: total (*n* = 51)	Interview participants^a^ (*n* = 10)
Gender				
Female	17 (55)	16 (80)	33 (65)	5 (50)
Male	14 (45)	1 (5)	15 (29)	5 (50)
Other	0 (0)	3 (15)	3 (6)	0 (0)
Age (years)				
0–2	10 (32)	0 (0)	10 (20)	0 (0)
3–5	11 (36)	0 (0)	11 (21)	5 (50)
6–8	3 (10)	0 (0)	3 (6)	2 (20)
9–12	1 (3)	0 (0)	1 (2)	1 (10)
13–16	6 (19)	20 (100)	26 (51)	2 (20)
Region of UK				
England	24 (77)	12 (60)	36 (70)	9 (90)
Northern Ireland	2 (6)	0 (0)	2 (4)	0 (0)
Scotland	3 (10)	6 (30)	9 (18)	0 (0)
Wales	2 (6)	2 (10)	4 (8)	1 (10)
Ethnicity^b^				
Arab	0 (0)	0 (0)	0 (0)	0 (0)
Asian or Asian British	4 (13)	1 (5)	5 (10)	1 (10)
Black, Black British, Caribbean or African	0 (0)	0 (0)	0 (0)	0 (0)
Mixed or multiple ethnic groups	3 (10)	0 (0)	3 (6)	1 (10)
White	24 (77)	18 (90)	42 (82)	7 (70)
Other^c^	0 (0)	1 (5)	1 (2)	1 (10)
Disability	4 (13)	2 (10)	6 (12)	1 (10)
Number of years under dermatology				
<1	5 (16)	2 (10)	7 (14)	0
1–2	12 (39)	1 (5)	13 (25)	0
2–3	5 (16)	2 (10)	7 (14)	4 (40)
>3	9 (29)	15 (75)	24 (47)	6 (60)
Treatments				
Topical	31 (100)	20 (100)	51 (100)	10 (100)
Phototherapy	0 (0)	2 (10)	2 (4)	0 (0)
Systemic	2 (7)	6 (30)	8 (16)	1 (10)

Data are presented as *n* (%). ^a^Demographics relate to the patient (child or adolescent) and not the parent. ^b^Ethnicity was self-described for the interviews. For the survey, participants chose from predefined census categories. If they selected ‘Other’, participants were asked to type in their exact ethnicity. ^c^Latin American.

Interviews were conducted on Microsoft Teams using a semi-structured topic guide (Appendix S2; see Supporting Information). Consent was reconfirmed verbally before each interview. Interviews were conducted by N.K.S. and A.D., who are trained in qualitative interviewing. N.K.S. and A.D. are clinicians, which may have introduced researcher bias compared with non medical interviewers. However, participants were not patients known to the interviewers, and their clinical knowledge of eczema and the NHS was beneficial in achieving a depth of discussion to meet the study objectives. A qualitative researcher (J.M.) supervised the interview study. Initial interviews were discussed (N.K.S., A.D. and J.M.) and topic guides adapted iteratively as interviews proceeded. Mean interview duration was 59 min. Interviews were transcribed verbatim and managed using QSR NVivo 12 software. Transcripts were checked for accuracy by N.K.S. and re-read to become familiar with the data.

Data were analysed thematically using framework ­analysis, a method developed by Ritchie and Spencer in the 1980s for large-scale policy research, now increasingly used in health research.^16^ This was chosen due to its systematic approach supporting team collaboration. Initial transcripts were read and coded independently by N.K.S., A.D., S.M. and J.M. Codes were discussed and agreed upon to form an initial analytical framework. Codes that were conceptually related were grouped to form categories. The framework was used to code subsequent transcripts and revised to incorporate new or refined codes. No new codes or changes to the index were required after nine interviews. The final analytical framework was used to index all transcripts using NVivo 12. Data were then inputted into a matrix using Microsoft Excel. This comprised one row per participant and one column per code, with a separate sheet for each category. The matrix was reviewed and themes generated by inductively identifying patterns and links across and within codes and cases. Analytical memos were used to explore the data and generate possible explanations. Final themes and subthemes were defined by the research team (N.K.S., A.D., S.M., E.B.-T. and J.M.), with a focus on addressing the study objectives.

## Results

### Online survey

We received 59 responses, of which 8 were excluded as it was apparent that the participant had not had a teleconsultation, despite indicating so in the eligibility questions. A total of 31 parents and 20 adolescents from across the UK were included in the analysis. Table 1 shows patient demographics.

### Experiences of and preferences for eczema follow-up

**Figure 1:**
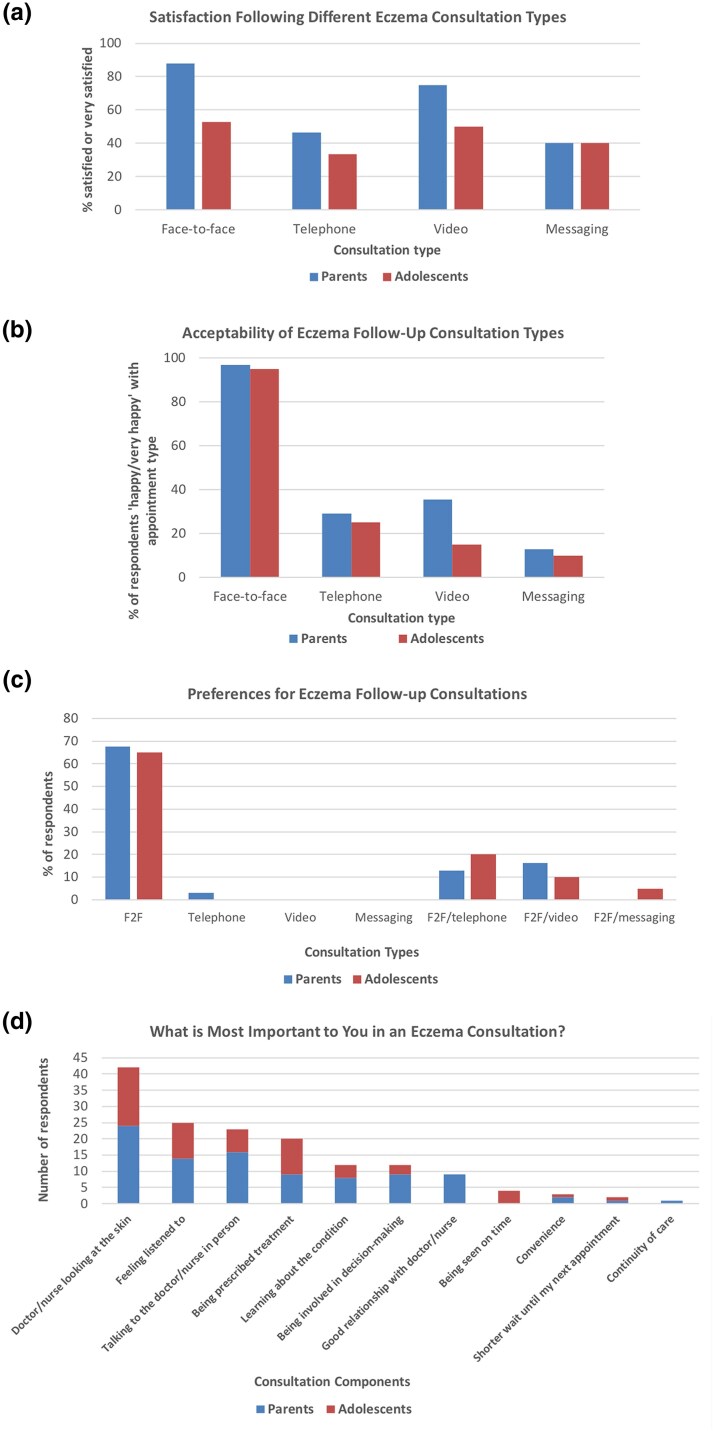
(a) Percentage of respondents who were satisfied or very who were satisfied following different types of eczema consultation. (b) Percentage of respondents who were happy or very happy with each appointment type for their next follow-up appointment. (c) Preferences of appointment type for eczema follow-up. (d) Most important aspects of an eczema consultation. F2F, face-to-face.

Appointment types experienced by patients included face-to-face (82%), telephone, (92%), video (28%) and online or mobile messaging (26%). Most participants (88%) had no technical problems during their teleconsultation. Those who did predominantly reported issues with poor connection. Of those who had experienced face-to-face appointments, 74% overall were ‘satisfied or very satisfied’ with their appointment vs. 41% for telephone, 71% for video and 40% for online or mobile messaging (Figure 1a). Dissatisfaction was reported most frequently following telephone appointments (33%). Free-text responses clarified reasons for this including:

‘Didn't feel listened to. Clear time limit 4 mins. Felt was not treated as an individual just tick box onto next cream.’ (Parent of 2-year-old girl)

‘The doctor failed to diagnose eczema herpeticum from the photos sent.’ (Parent of 14-year-old girl)

Of all participants, 96% reported they would feel ‘happy or very happy’ if their next appointment was face-to-face vs. 28% for telephone, 28% for video and 12% for online or mobile messaging (Figure 1b). When asked about preferences, 67% of all participants’ first-choice consultation was face-to-face, followed by 16% preferring a combination of face-to-face and telephone and 14% preferring a combination of face-to-face and video (Figure 1c). Parents and adolescents preferred face-to-face appointments. Reasons for preferred consultation type are shown in Table 2.

**Table 2:** Illustrative quotations from survey respondents: reasons for preferred type of follow-up appointment

Preferred consultation type	Reasons for preference	Participant
Face-to-face	‘Anything other than face-to-face feels like a waste of time. The doctor can't see how bad my child's skin is over the telephone!’	Parent of 16-year-old boy
	‘Because sometimes it is about skin texture, which can't be described over the phone and needs to be seen in person.’	Parent of <1 year boy
	‘I chose face-to-face because I don't think anyone can get the gist of the true scale of the problem over phone or video. I think some follow-up appointments can be done this way; however, this isn't a good process to use when getting to know a patient's situation.’	Parent of 15-year-old boy
	‘…I’m more likely to be more descriptive about certain aspects of my skin condition face-to-face with my doctor compared to on the telephone or email/messages.’	15-year-old girl
	‘I think that it's better for the doctors to see how bad the eczema actually is, which can determine how serious the issue actually is. I would rather have a face-to-face appointment because I find it more simple to explain how the eczema is in person.’	16-year-old girl
Mix of face-to-face and telephone	‘When my child's eczema is bad or not responding to her treatment plan I like to have a face-to-face to check the skin for a thorough assessment of her current treatment plan. However, when my daughter's skin is being well managed the practicalities of taking a toddler to the hospital for a quick “great it's all working” is challenging. So, in these cases I find a phone call would be much easier for us.’	Parent of 2-year-old girl
	‘Sometimes really hard to get days off for appointments. Being self-employed means not being able to take on work.’	Parent of 13-year-old girl
	‘Doctor able to see your skin and how it's improved or worsened but can also speak to you over the phone to discuss how treatment is going for you. Not always having to go to appointments and missing school, etc.’	16-year-old girl
Mix of face-to-face and video	‘Face-to-face is ideal but now my daughter is in school it's trickier. Video appointments are ideal as I don't have to park at the hospital or book as much time off, but face-to-face is still needed at times to be properly looked over.’	Parent of 4-year-old girl
	‘Convenient by video as dermatologist over an hour away. But sometimes good to see the skin.’	Parent of 4-year-old boy
	‘It is much easier to keep on top of my eczema with face-to-face appointments as the texture of skin will sometimes get worse with some medications or get better and the having the choice of occasional video calls would mean that the dermatologist has an accurate image of the skin itself and it helps with if I have prior commitments so I can just take the call rather than missing out.’	16-year-old girl

Participants were asked what is most important to them in a consultation (Figure 1d). Only 6% of all respondents felt their top three consultation priorities could be met through a telephone appointment, while 49% felt they could be partially met. For video appointments, 16% felt their priorities could be met and 59% felt they could be partially met. Participants were asked about costs associated with attending different appointment types, but due to incomplete responses these data are not presented.

### Willingness to take part in a trial

Participants were informed about a potential randomized controlled trial comparing paediatric eczema follow-up by teleconsultation with face-to-face appointments. Most thought that telephone (84%) and video (80%) appointments should be included in the study. The majority of respondents (94%) would be happy or very happy to be randomized to the face-to-face group vs. 31% for telephone, 41% for video and 8% for online or mobile messaging. When asked about participating in a potential study, 41% were interested, 29% were unsure and 29% were not interested.

### Interviews

**Figure 2:**
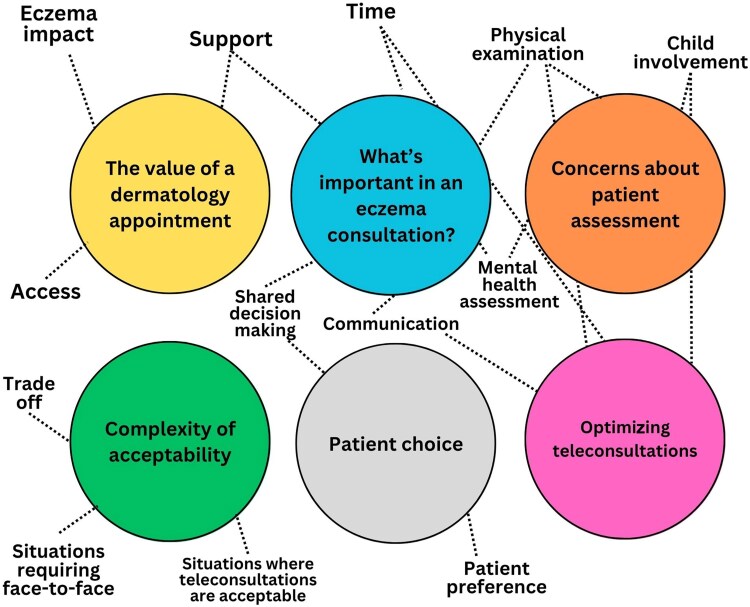
Schematic of themes.

We interviewed 12 participants: 8 parents (participants P01–P08) and 2 adolescent-parent dyads (participants A01/P09 and A02/P10). Patient demographics are provided in Table 1. Six overarching themes were constructed (Figure 2).

### Theme 1: the value of a dermatology appointment

Participants were unanimous in stating that eczema had a significant impact on the child or adolescent, highlighting impacts, including disturbed sleep, pain and reduced confidence. Parents and adolescents felt that eczema was more than just a skin condition and deserved to be taken seriously by healthcare professionals:

‘…you think of it “oh, it's just eczema, it's just a bit of dry skin”, and I didn't realise how much it would take over every aspect of my life completely.’ (P02)

Parents felt isolated, unsupported and overwhelmed by the self-management of their child's eczema, while dealing with sleep deprivation and the impact on their child and family. The dermatology appointment was a source of support, a place to vent and feel listened to. Much responsibility was felt in self-managing their child's eczema; therefore, they valued the opportunity for education and reassurance:

‘I know that the doctor is going to say on Wednesday “Do X number of days of steroid cream on him, and then do Protopic, and then try every other day”, but I almost want to go there, them to look at it and then to say it again, because we do so much maintenance it's quite overwhelming. We’ve been doing this for 2 years, and you almost want someone to say, “Yeah you are doing it correct”’ (P01)

Participants experienced delays accessing dermatology, long waits between appointments and unexpected cancellations. One participant resorted to private appointments due to lack of local services:

‘Basically, we’ve had to go private, because we’ve been told there's no paediatric dermatology services in the county we live in.’ (P06)

Collectively, these factors placed a high value on each dermatology encounter and provide a context for how parents and adolescents felt about different appointment types and what was acceptable to them (Table 3).

**Table 3:** Interview data demonstrating the value of a dermatology appointment (theme 1)

**Theme 1: the value of a dermatology appointment**		
Eczema impact	‘So I think it's not like a bit of dried skin, this is something that every day affects us, and it is a real… it's not like a tiny rash that I’m worried “Oh no, he's got a rash, it doesn't look perfect”, it's that he's got uncomfortably inflamed, I can't wash him. He couldn't walk.’	P01
	‘So, in that very difficult [time] of zero sleep and just pain and infections, they [dermatology] were extremely helpful, so invaluable.’	P04
Support	‘You want to have your struggle as the parent acknowledged as well, that it's not easy for you either, that yeah it's not counselling for me in the fact that I find it difficult, but just having that acknowledged is really helpful.’	P03
	‘I think it feels quite isolating maybe being a eczema parent and just struggling with the sleep deprivation, and the itching and all of that. So sometimes when we’re just talking to someone and they’re listening it doesn't really matter if it's face-to-face or telephone, you feel a bit in the moment it's quite satisfying I guess to get everything off your chest and feel like someone's helping you.’	P06
Accessing dermatology	‘I keep asking the GP about going to dermatology, they weren't really very keen to refer her, even though it was clearly not being well managed.’	P03
	‘…they were limiting the amount of referrals they were able to make, and yeah it took a long time of basically begging for an appointment to then at an off chance getting through to a different GP who immediately put the paperwork through.’	P04

### Theme 2: what is important in an eczema consultation

Good communication skills were essential, with participants reporting that feeling listened to was most important to them in an appointment (Table 4). Some shared experiences of clinicians who had communicated poorly, being dismissive or abrupt.

‘I was spoken down to a lot and I wasn't heard.’ (P08)

Effective communication was critical to the success of ­teleconsultations, particularly via telephone where body language and facial expressions were not visible. Participants felt less able to open-up on the telephone, particularly about their well being. This was mitigated where there was an existing clinician–patient relationship prior to the teleconsultation. Adolescents felt more involved in face-to-face appointments and children valued the opportunity to see other children with eczema in the waiting room:

‘I remember making friends with eczema. Even just going and playing with the toys and seeing all the doctors and nurses, because obviously I went there often so I felt like a celebrity going, everyone would be like, “Hello, how are you? How are you doing?” I loved it.’ (A01)

‘…something I hadn't even thought about was that she liked going to the appointments because you saw other children waiting who also had the same condition.’ (P02)

Participants wanted shared decision-making and more involvement in their or their child's care. Time was another important factor. Some felt that teleconsultations were shorter and rushed, whereas others felt they had more time. One parent with disabilities found communication by telephone difficult and highlighted that disabilities were never considered by the department when issuing ­teleconsultations.

**Table 4:** Interview data illustrating what is important in a dermatology appointment (theme 2)

**Theme 2: what is important in a dermatology appointment?**		
Communication	‘For me it's just that you’re listened to really.’	P09
	‘Yeah, I can be really shy, so making sure that they actually talk to me rather than just my mum.’	A01
	‘She likes the appointments if the consultant is friendly and speaks to her as a person rather than speaking to me.’	P05
	‘I think communication skills need to be up to scratch if you are doing these teleconsults, because that's the real thing where people are left really unsatisfied.’	P08
Time	‘In fact, to be honest I felt that all of our phone consultations felt less rushed. I don't know, it might be the lack of body language, but you could sense that on the face-to-face appointments, you could sense the urgency of “I’ve got other patients to look at, could you please leave”, whereas on the phone, maybe it's also them being too kind to say, “Look, can you just stop asking questions”, but there was definitely times that I knew that I was definitely taking a lot of time.’	P03
	‘So, appointments were very much shorter as well, because they were just phone calls, and I didn't feel like I was in control at all during those appointments, because when you’re in a face-to-face appointment the other person can't shoo you out the door, here they can just put down the phone.’	P07
	‘…they are very fast, they see her skin and they prescribe creams and that's it.’	P10
Shared decision-making	‘… and it being a two-way discussion, not just “Do this, off you go”. Talking, problem solving it together, what's been going well, and then trying to pick apart if it's not been working then why not.’	P03
	‘She said before she started the conversation that, “I would like to discharge you during this session”, so it wouldn't have mattered much what I had said.’	P07
	‘I think a good appointment would be for as a parent being heard, them understanding your concerns and the distress of your child, and working together to come up with the best management plan.’	P08

### Theme 3: concerns about patient assessment

Skin examination was considered a key part of the consultation and concerns were raised about this aspect of teleconsultations. Most felt that photographs were unable to capture eczema accurately, particularly in skin of colour, and that examining skin texture was important.

‘Also, my son is mixed race so again different sort of shade of skin, things also appear differently on it, so I felt I was worried definitely whether that's going to come across in pictures.’ (P04)

However, some participants liked being able to choose which photographs to send, having had experiences where their child's eczema had cleared on the day of their appointment. Photographs were deemed more accurate than video examination. With both modalities, it was difficult to encourage young children to cooperate, while older children did not always feel comfortable.

‘…there's eczema all the way up to the inside of my thigh and stuff, and I wouldn't have liked a photo there or anything.’ (A01)

Most concern was surrounding telephone consultations where no option was given to send photographs at all. Parents felt it was unsafe to prescribe treatment based on their verbal description alone.

‘…it was based on my judgement really, and I would have felt better if it was more of an inspection.’ (P04)

Lack of involvement of the child or adolescent was another concern with teleconsultations, with missed opportunities for assessing mental health, growth and development. Adolescents felt left out of telephone appointments. Involving young children, who could not sit still for a video appointment or speak on the telephone in teleconsultations was particularly challenging. Most participants felt face-to-face appointments were required for younger children, with children aged >8 years more likely to be able to participate in video consultations (Table 5).

**Table 5:** Interview data illustrating concerns about patient assessment (theme 3)

**Theme 3: concerns about patient assessment**		
Physical examination	‘…I remember taking the photos and it didn't look too bad compared to what I actually had.’	A02
	‘But I think we were able to take an okay picture. I think they were able to assess it quite well from the photo.’	P01
	‘I was asked to assess the skin, but not in any detail, just in the basic better or worse.’	P07
	‘The thing about seeing them face-to-face is looking at the skin and looking at any negative effects of the steroids, like thinning of the skin.’	P08
Mental health assessment	‘Because I think sometimes you can't express… it's the little things isn't it? Like the facial expressions from a child that you’re not going to get in a photo.’	P05
	‘…you do pick up a lot more seeing somebody's face than just hearing their voice. You can hear it in their voice sometimes, but people can hide it a lot better.’	P09
Involvement of the child	‘…for [daughter] it makes it feel like we are paying attention, we do know we are listening to her. Here is somebody that's seeing it. Whereas I suppose if I just say I’m on the phone she doesn't necessarily see that input so much.’	P02
	‘…the face-to-face ones were more stressful for my child, because he was involved; he was not involved in the telephone appointments, and then being born into COVID and then having to go into hospital with many people around him and having to get weighed or things like that, that… yeah didn't always go well.’	P04
	‘I couldn't really hear much about what was going on unless my mum put them on the phone to me, where again I still didn't feel quite as comfortable, which I didn't like.’	A01
	‘I’d say the toddler age is not ideal… for any type of call, but I think your study is more for older children as well I guess, 8 or 9 or something, and maybe they can engage a bit more and they can show their itchy areas to the phone and stuff like that a bit more.’	P06

### Theme 4: complexity of acceptability

Whether participants found teleconsultations acceptable was a complex entity. While not a replacement for face-to-face appointments, they had value in certain situations. For example, if the eczema was stable and an effective treatment plan in place, teleconsultations were considered a convenient option that would reduce time missed from work and school. They were also a valuable alternative to avoid cancelling an appointment if they could not attend in person, such as having chickenpox. Some felt teleconsultations would be more acceptable if it meant having more frequent contact with a clinician, to bridge the wait between face-to-face appointments:

‘…if teleconsultations would be added to what was already given…the normal appointments kept and then added teleconsultations occasionally to check-up.’ (P07)

Participants agreed that face-to-face appointments were essential for first consultations, uncontrolled eczema and infections. They were most valuable early on in their eczema journey, when education and support were particularly important. Face-to-face was also deemed necessary where there were safeguarding issues, need for language interpretation or disabilities that would impair communication via teleconsultation (Table 6).

**Table 6:** Interview data on the complexity of acceptability (theme 4)

**Theme 4: the complexity of acceptability**		
Situations where teleconsultations are acceptable	‘I think they’re almost for different things. I think for in-between appointments I think they’re really useful.’	P01
	‘If actually we felt like we were managing the skin, and the plan was good, and it was all going well, then actually a telephone consultation I think is fine.’	P02
	‘So, if it was going to fall on a really… I don't know, like an important week at school, and I didn't want her to miss it, or I don't know, or just if I was really busy with work, then a video one would just be great, it would just be a lot easier to fit in.’	P03
	‘I think if nothing significant has occurred since the last appointment I would feel comfortable with a telephone conversation.’	P04
	‘…obviously appointments are during working hours, to get to the hospital that's a whole morning off and a whole morning off of school, so whereas I think if there was a telecommunication every now and then that would only be half an hour rather than a few hours.’	P05
	‘So, I would probably say face-to-face if things aren't going well, and telephone if things are alright, because they do just generally with eczema there are moments where it's a lot better, and then it's a waste of everyone's time to just see it when things are fine.’	P08
	‘Telephone appointments maybe in COVID times was good, or when she broke her leg as well.’	P10
Situations that require face-to-face	‘But if you’re having relentless infections, and you feel like the skin you aren't managing it and you can't get on top of it, and they’re in constant pain, actually I think going to see someone is much more valuable, and also for the child, for [daughter] it makes it feel like we are paying attention, we do know we are listening to her. Here is somebody that's seeing it.’	P02
	‘I think I would always choose face-to-face for an initial appointment.’	P03
	‘I feel like the initial assessment should always be in person, so you can fully gauge the child and how severe the problem is, and then thereafter I guess some children are more appropriate for a telephone appointment.’	P08
	‘I think you need to see them in person the first few times you see them definitely.’	A01
	‘If there were safeguarding issues for a child, if they’re not…if they’re having repeated telephone consultations for that child I don't think that's ideal, because they need to physically lay eyes on them at some point.’	P03

### Theme 5: patient choice

When asked about their preferences for modality of follow-up, this varied, with some preferring exclusively face-to-face appointments and others preferring a mix of face-to-face and teleconsultations. Nobody felt that follow-up exclusively by teleconsultation was acceptable. All participants would value more choice in deciding how they, or their child, are seen. They felt it should be a shared decision, accounting for age, eczema severity and the ability of the child to participate in a teleconsultation (Table 7). Due to the relapsing–remitting nature of eczema, it was felt there should be flexibility to change the appointment type nearer the time:

**‘**…if there was a way of working out a check-in 2 weeks prior to the meeting to see whether…maybe setting it as a phone one and checking in 2 weeks prior to that, are you still happy with it being phone or would you rather it was face-to-face.’ (P04)

**Table 7:** Interview data on patient choice (theme 5)

**Theme 5: patient choice**		
Patient preference of eczema follow-ups	‘I’d probably always prefer to go in person.’	A02
	‘I think I’d want to know there was a follow-up with face-to-face. But I think 2 weeks maybe, 2 or 3 weeks after you’ve had your face-to-face appointment to have a telephone one set in would be I think really useful.’	P01
	‘I think I would always choose face-to-face for an initial appointment, and then I would be between video and face-to-face later.’	P03
	‘Maybe if my skin's really flaring then I’d want to see them. But if it's…maybe a mix of both, either the video call and messaging photos, so it's like you message photos then you talk about it over the video call and stuff like that.’	A01
Deciding how a patient is followed-up	‘But I think if you could have the choice then I think that would be good. But I think there's been times when we’ve just been, “Your appointment is on the phone,” that we felt a bit “Oh, we wanted to see someone”.’	P01
	‘I would love it if they ask you if you felt he needs to be seen face-to-face, because it's not like parents want to waste a dermatologist appointment, they wouldn't want to waste their petrol, their time, childcare, all that stuff unnecessarily, they’d only do it if they felt they really needed.’	P08
	‘I think that something like some sort of an assessment should be made, like a questionnaire or that sort of thing, in which disabilities, accessibility, ability to communicate and preferences are all taken into account, and then something decided on based on that.’	P07

### Theme 6: optimizing teleconsultations

Participants felt teleconsultations could be improved if conducted by a clinician they knew, who had examined their or their child's skin before. There needed to be enough time to describe the skin, address concerns and write down the treatment plan, as there were often delays receiving clinic letters. Photographs should be sent before the appointment to make the process more streamlined. Participants would value instructions on taking effective photographs, acknowledging different skin tones. It was felt that clinician communication skills could be improved, particularly for telephone consultations, with the suggestion of training (Table 8).

**Table 8:** Interview data on optimizing teleconsultations (theme 6)

**Theme 6: optimizing teleconsultations**		
How could teleconsultations be improved?	‘I think if you can send the pictures first I think then so the doctor can look at them before and then give you a call.’	P01
	‘…giving you that time to be able to write down the plan yourself, and being able to go over and it clarify the information.’	P02
	‘Really clear questions that are easy to answer.’	P03
	‘…definitely send a request, like an active request for photos from the practitioners, and possibly with any instructions or if there's any lessons learnt from…that they might have from experience on even taking the photos and acknowledging the different skins types and skin colours as well, that would have probably been helpful, is it better to do it in daylight, or night time but with a strong light, or maybe no light, or at an angle.’	P04
	‘No, because without being blunt it is what it is.’	P05
	‘I think your clinicians need to be trained well for them in terms of communication skills.’	P08
	‘Making sure you can send photos before the call so they know what you’re talking about.’	A01

## Discussion

Our study provides an insight into the experiences and preferences of adolescents and parents having teleconsultations for eczema follow-up in secondary and tertiary care. There were low levels of satisfaction following telephone appointments (41%), with higher levels of satisfaction following face-to-face (74%) and video (71%) appointments. Dissatisfaction with teleconsultations was predominantly due to lack of skin examination and communication difficulties, both of particular concern with telephone appointments. Parents and patients highly valued their dermatology appointments, often enduring long waiting times, resulting in a preference for face-to-face appointments. However, some preferred the option of a combination of face-to-face and teleconsultations. Acceptability of teleconsultations was complex, with a trade-off between convenience vs. the need for an in-person skin examination and other benefits of face-to-face appointments such as a more personal encounter.

Previous studies within paediatric dermatology showed a trend towards higher satisfaction following video consultations than telephone; however, most were service evaluations and these modalities have not been compared in a trial.^17–21^ It should be noted that most of our survey participants had experienced telephone consultations (92%), associated with higher levels of dissatisfaction, compared with video consultations (28%), which may have contributed to the preference for face-to-face appointments within our sample. Our results were in concordance with a service evaluation of paediatric dermatology telephone consultations during the pandemic, where 65% of patients and parents preferred to be seen face-to-face in future appointments.^19^

Despite the preference for face-to-face appointments, just under a third of survey participants preferred a combination of appointment types for follow-up. Acceptability of tele­consultations was dependent on variable factors, including eczema severity and stability, work and school commitments and whether they would be conducted with a clinician known to them. These findings are similar to studies conducted within other paediatric specialties. A Welsh study of general paediatric telephone clinics found teleconsultations were more acceptable to parents for uncomplicated follow-up appointments, where a clinical examination was not required.^22^ There was less acceptability for ongoing telephone appointments in younger children <2 years. A Canadian qualitative study of adolescents and caregivers having video consultations within paediatric nephrology also found that teleconsultations were preferred for routine follow-ups, but that face-to-face was preferable when there was a change in clinical condition or for more complex disease.^23^

Pressure on NHS waiting lists continues to rise; the dermatology waiting list in December 2023 had increased by 86% since January 2020.^24^ Waits for non cancerous skin disease are particularly long due to prioritization of skin cancer referrals.^6^ The use of teleconsultations within dermatology has reduced dramatically since the COVID-19 pandemic, with a 61% reduction in follow-up teleconsultations from 2020–21 to 2023–24.^25,26^ This likely reflects the preferences of patients and clinicians and the need for physical examination in skin conditions.^14,27^ The British Association of Dermatologists Lessons Learnt Questionnaire in 2020 found video consultations were poorly received by clinicians due to poor diagnostic quality and efficiency, noting that remote reviews could take longer and may simply defer a face-to-face appointment.^28^ However, offering teleconsultations for suitable patients has many potential benefits, including increased access to appointments, reduced missed appointments and reduced environmental impact.^27^ Remote consultations are a key component of the NHS Outpatient Recovery and Transformation Programme and the NHS commitment to net zero by 2040.^4,29,30^

Study participants felt teleconsultations could be improved by being conducted with a clinician known to them and better communication. Providing safe and effective teleconsultations is now part of the UK dermatology training curriculum.^31^ Interview participants wanted guidance on taking photographs and highlighted that these should be sent before the appointment. Patient guidance now exists on taking photographs effectively for a teleconsultation.^32,33^ Participants also raised concerns that eczema severity may not be reflected accurately in a photograph. Studies investigating THE accuracy of remote eczema severity assessment found high concordance between assessment via photographs and in person. However, limitations included not using patient-provided photographs, lack of skin tone diversity, not including children and not measuring inter- and intra-rater reliability.^34–37^ Croce *et al.* investigated the validation of remote eczema assessment in children using caregiver photographs and videos, finding high concordance with in-person assessment for photographs and moderate-to-high concordance for video.^38^ Future remote eczema severity assessment may be aided by artificial intelligence-powered image analysis. There has been rapid development in this field, yet current models are limited by insufficient datasets.^39,40^

Another key concern with paediatric teleconsultations was the lack of involvement of the patient. Adolescents felt left out of telephone consultations, while video consultations were unsuitable for young children. The impact on the quality of life and psychological health of children and adolescents with eczema is well documented.^41,42^ In a qualitative study involving adolescents with eczema, key messages were to address the emotional impact, give more information and take their condition seriously.^41^ The British Society for Paediatric and Adolescent Dermatology published recommendations on mental health assessment and support in children and adolescents with skin conditions.^43^ Recommendations to assess the emotional impact of skin conditions and screen for quality of life and mental health impacts may be challenging to implement remotely, particularly offering adolescents the opportunity to be seen alone and recognizing nonverbal signs such as low affect. A survey of adolescents with chronic fatigue syndrome having teleconsultations found that only 36% had the opportunity to speak to their clinician alone.^44^

The strengths of this study include its mixed-methods approach and the inclusion of adolescents and parents. The survey captured a broad range of experiences and views of teleconsultations, which were explored in greater depth through qualitative interviews. Our decision to recruit participants online had the advantage of obtaining a heterogeneous sample of patients and parents from across the UK, compared with other studies, which tend to be limited to single centres. However, this did make it challenging to reach our target population. Our study is limited by a small sample size for both survey and interviews, with under-representation of Black and adolescent male patients. Recruiting online may have also excluded certain groups, who may be vulnerable to digital exclusion through teleconsultations, such as those without internet access or who do not speak English. Previous US studies showed lower uptake of paediatric teledermatology visits among Black patients and those for whom English was not their first language.^45^

This study aimed to inform a future trial to assess the effectiveness and acceptability of teleconsultations for paediatric eczema follow-up. Our findings indicate that a study intervention comprising exclusively teleconsultations would not be acceptable to patients or parents. A more acceptable intervention would be a hybrid approach, incorporating face-to-face and teleconsultations, with the flexibility to change the appointment type. Increasingly, NHS trusts are offering more flexible follow-up options, such as patient-initiated follow-up, aiming to reduce pressure on waiting lists by arranging appointments when clinically needed. These flexible approaches make designing a study intervention challenging, in addition to the differences in service delivery between trusts, changing healthcare policy and rapid development of technology. The implications of our study for clinical practice are the insights into what patients and parents value about dermatology appointments. Being listened to, examination of the skin and the opportunity to make shared decisions were key components of an effective consultation. We have also shared ways that teleconsultations can be delivered more effectively, from a patient and parent perspective. Departments and clinicians should offer parents and patients more choice and flexibility on the modality of their appointment, taking a more personalized approach, in line with NHS strategy.

## Supplementary Material

vzaf123_Supplementary_Data

## Data Availability

The data underlying this article will be shared on reasonable request to the corresponding author.
