# Supplementary material for: Teleconsultations for Eczema in CHildren (TECH) feasibility study: a mixed-methods study with adolescents and parents
Source: Skin Health Dis. 2026 Jan 30;6(2):125–37. doi: 10.1093/skinhd/vzaf123 (PMC13036726; doi:10.1093/skinhd/vzaf123)
Supplement: vzaf123_Supplementary_Data [file vzaf123_supplementary_data.zip › Appendix S1.docx]

**
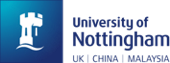

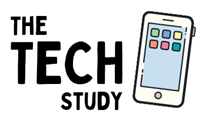
**

**Tell us about your telephone or video appointment for eczema!**

***PAGE 1***

**Welcome!**

**Thank you for your interest in our online survey! We want to hear from young people and parents who have had teleconsultations for follow-up of eczema in UK dermatology (skin) departments.**

***What is a teleconsultation?***

*A teleconsultation is an appointment given by phone, video or mobile apps, instead of coming to the hospital in person.*

***What do you mean by follow-up?***

*This is an appointment* ***after*** *your first appointment with your dermatology doctor.*

In this survey we will ask about how you find having teleconsultations for eczema. We want to hear about which types of appointments you prefer and we will also ask about how much money and time you spent with different appointment types.

The survey will take about 10 minutes to complete. Your answers will be kept anonymous (will not be linked to your name).

***PAGE 2***

**First we need to check if you have had the experiences we are looking for to answer the survey**

**1.**  **I am a young person (age 13-16 years) with eczema under the care of a UK dermatologist**

**Or**

**I am the parent of a child (age 0-16 years) with eczema under the care of a UK dermatologist**

- **Yes** – **proceed**
- **No** – thank you for your interest in our survey. Unfortunately in this survey we are only looking for participants who are under follow-up care by dermatology in the UK. **(Don’t proceed)**

**2.**  **I have had at least one dermatology *follow-up* appointment via telephone, video or mobile app for my/my child’s eczema**

- **Yes – proceed**
- **No** – thank you for your interest in our survey. Unfortunately in this survey we are only looking for participants who have had at least one dermatology teleconsultation follow-up appointment. **(Don’t proceed)**

**PAGE 3**

**Deciding whether to take part**

Before deciding whether or not to take part in the survey, please read the information sheet and give your agreement if you are happy to take part.

You can access the participant information sheet **here**

If you are under 16 years old you must ask a parent/guardian to read the information sheet and obtain permission to take part in the survey.

**(If consent form signed *(see Appendices 1 and 2)* 🡪 proceed to survey)**

**PAGE 4**

**Thanks for agreeing to take part! Firstly a few questions about you!**

***If you need help with answering any of the questions, please ask someone in your family or a carer***

**1. Are you a..**

- Young person
- Parent

**2. What is your gender?**

- Female
- Male
- Prefer to self-describe as _________
- Prefer not to say

**3. How old are you?**

___________

**4. What region of the UK do you live in?**

- England
- Northern Ireland
- Scotland
- Wales

**5. What is your ethnic background?**

- Arab
- Asian or Asian British
- Black, Black British, Caribbean or African
- Mixed or multiple ethnic groups
- White
- Other
- Prefer not to say

**6. Do you consider yourself to have a disability?**

- Yes
- No
- Prefer not to say

**7. Which of the following do you have access to and know how to use? (Tick all that apply)**

|  | **I have access to this** | **I know how to use this** |
| --- | --- | --- |
| Home telephone |  |  |
| Smartphone |  |  |
| Computer (such as laptop, tablet or desktop) with webcam |  |  |
| Broadband internet |  |  |

**8. How long have you been seeing a dermatologist about your eczema?**

- <1 year
- 1-2 years
- 2-3 years
- >3 years

**9. What treatments are you taking for your eczema?**

- Moisturisers
- Other creams prescribed by a doctor or nurse (such as steroid cream)
- Light treatment (phototherapy)
- Tablets or injections
- Other

**10. Do you see a dermatologist in the NHS or privately (tick all that apply)**

- NHS
- Private

**Page 6**

**Now moving onto teleconsultations – we want to hear about your experiences**

Please note in this survey we are only asking about your dermatology follow-up appointments and not teleconsultations you may have had with your GP or other hospital departments.

**1. What types of appointments have you had for follow-up of your eczema? (Tick all that apply)**

- Face-to-face
- Telephone (without photos)
- Telephone (and photos of my skin were looked at)
- Video (without photos)
- Video (and photos of my skin were looked at)
- Messaging via mobile app/online/email
- Other

**(Only if they tick with photos) -**

**1b. How did you send photos?**

- Email
- Mobile app
- Website or online portal
- Other _______________

**1c. Were you given instructions on how to take the photos?**

- Yes
- No

**2. Before your teleconsultation were you asked to send your doctor/nurse any of the following:**

- DLQI score (quality of life score)
- POEM score (eczema severity score)
- Diary of eczema flares
- Other _______________________________

**3. Did you experience any technical problems during your teleconsultation?**

- Yes
- No

**3b. If yes please tell us the type of teleconsultation and what problem you experienced**

____________________________________________________

**4. How satisfied were you with the care you received during these different appointments? *(Tick N/A if you haven’t had a particular type of appointment)***

|  | **Very** | **Somewhat** | **Neutral** | **Not very** | **Not at all** | **N/A** |
| --- | --- | --- | --- | --- | --- | --- |
| **Face-to-face** |  |  |  |  |  |  |
| **Telephone** |  |  |  |  |  |  |
| **Video** |  |  |  |  |  |  |
| **Mobile/online messaging** |  |  |  |  |  |  |

**4b. If no why? __________________________________________________________**

**PAGE 7**

**What is important to you when you see the doctor or nurse about your eczema?**

1. **If your next appointment for your eczema was one of the following, how happy would you be with this?**

|  | **Very happy** | **Quite happy** | **Neutral** | **Unhappy** | **Very unhappy** |
| --- | --- | --- | --- | --- | --- |
| **Telephone** |  |  |  |  |  |
| **Video** |  |  |  |  |  |
| **Mobile/online messaging** |  |  |  |  |  |
| **Face-to-face** |  |  |  |  |  |

1. **If you had the choice how would you like to be seen in dermatology for your eczema? (Please rank in order of preference)**

- Face-to-face only
- Telephone only
- Video only
- Mobile/online messaging only
- Mix of face-to-face appointments and telephone
- Mix of face-to-face appointments and video
- Mix of face-to-face appointments and mobile/online messaging

**2b. Why did you choose your top ranked appointment? Any other thoughts about how you would like to be seen in dermatology?**

**____________________________________**

**3. What is most important to you when you see the doctor or nurse about your eczema? (Please tick the 3 most important from the list or add your own)**

- Talking to the doctor or nurse in person
- The doctor or nurse looking at my skin
- Convenience
- Being seen for my appointment on time
- Good relationship with the doctor or nurse
- Feeling listened to
- Being involved in decision-making
- Being prescribed treatment
- Learning about my condition
- Shorter wait time until my next appointment
- Other _________________________________

**4. Do you think your top 3 can be met through a telephone appointment?**

- Yes
- No
- Partially

**5. Do you think your top 3 can be met through a video appointment?**

- Yes
- No
- Partially

**6. Do you think your top 3 can be met through mobile/online messaging?**

- Yes
- No
- Partially

**PAGE 8**

**We understand that different appointment types carry different costs for patients. We want to hear about your experiences.**

***Some of these questions will ask about costs experienced by parents and carers, so please ask them or select the option for not known.***

1. **How would you usually travel to the hospital for a face-to-face appointment?**

- Car
- Bus
- Walk
- Cycle
- Train/tube
- Other

1. **How far do you travel to the hospital for a face-to-face appointment?**

- _____ (miles)
- Not known

1. **What costs have you or your family experienced when attending a face-to-face appointment? (Tick all that apply)**

- Travel
- Parking
- Childcare
- Loss of income
- Other
- Not known

**3b. If you are able to, can you estimate how much it costs you to attend a face-to-face appointment? (Optional)**

- £__________
- Not known

1. **Have you ever claimed back travel costs to and from your appointment through the ‘NHS Healthcare Travel Costs Scheme?’**

- Yes
- No

1. **What costs have you or your family experienced when attending a telephone appointment? (Tick all that apply)**

- Childcare
- Loss of income
- Travel to collect prescription
- Other
- N/A
- Not known

**5b. If you are able to, can you estimate how much it costs you to attend a telephone appointment? (Optional)**

- £­­­­­­­­­­­_________
- Not known

1. **What financial costs have you or your family experienced when attending a video appointment? (Tick all that apply)**

- Childcare
- Loss of income
- Travel to collect prescription
- Data or internet usage
- Other
- N/A
- Not known

**6b.** **If you are able to, can you estimate how much it costs you to attend a video appointment? (Optional)**

- £­­­­­­­­­­­_________
- Not known

1. **How much time off school do you typically miss with different types of appointment?**

|  | Don’t attend – my parent does | <1 hour | 1-2 hours | 2-3 hours | 3-4 hours | >4 hours | N/A |
| --- | --- | --- | --- | --- | --- | --- | --- |
| Face-to-face |  |  |  |  |  |  |  |
| Telephone |  |  |  |  |  |  |  |
| Video |  |  |  |  |  |  |  |

1. **How much time off work do you typically miss taking your child to their appointment?**

|  | None – I don’t work | <1 hour | 1-2 hours | 2-3 hours | 3-4 hours | >4 hours | N/A |
| --- | --- | --- | --- | --- | --- | --- | --- |
| Face-to-face |  |  |  |  |  |  |  |
| Telephone |  |  |  |  |  |  |  |
| Video |  |  |  |  |  |  |  |

**8b. When you miss work to attend your child’s appointment, how is the leave taken?**

- Unpaid leave
- Annual leave
- Paid leave
- Flexibly – I make up the time

**Page 9**

**Last few questions now! We want to hear your thoughts on a future research study comparing teleconsultations and face-to-face appointments for eczema**

***We are designing a clinical trial that would compare teleconsultations with face-to-face consultations for children with eczema. In this study some patients would be followed up by teleconsultations and some would be followed up by face-to-face appointment. Patients would not get to choose which consultation type they were allocated – this would be done randomly.***

1. **What type of teleconsultation do you think should be included in the study? (Tick all that apply)**

- Telephone
- Video
- Mobile/online messaging
- Other

1. **How would you feel about being randomly allocated to the following appointment types:**

|  | **Very happy** | **Happy** | **Neutral** | **Unhappy** | **Very unhappy** |
| --- | --- | --- | --- | --- | --- |
| **Face-to-face** |  |  |  |  |  |
| **Telephone** |  |  |  |  |  |
| **Video** |  |  |  |  |  |
| **Mobile/online messaging** |  |  |  |  |  |

1. **Is this a study you would be interested to take part in?**

- Yes
- No
- Not sure

**4. Is there anything else you would like to tell us about your experiences of teleconsultations for follow-up of eczema, that we haven’t covered?** *(Free text)*

_______________________________________________________________________
