# Supplementary material for: Teleconsultations for Eczema in CHildren (TECH) feasibility study: a mixed-methods study with adolescents and parents
Source: Skin Health Dis. 2026 Jan 30;6(2):125–37. doi: 10.1093/skinhd/vzaf123 (PMC13036726; doi:10.1093/skinhd/vzaf123)
Supplement: vzaf123_Supplementary_Data [file vzaf123_supplementary_data.zip › Appendix S2.docx]

**Exploring Patient Experiences and Views of Teleconsultations for Paediatric Eczema: A Qualitative Interview Study**

**Interview Topic Guide**

**Section 1: Establishing rapport**

- Getting to know you – where they are from, about their family, interests/hobbies/reason they were interested in taking part
- Explore their dermatology journey – how long they have been under the care of dermatology for eczema, what types of consultations they have experience of that we can talk about in the interview

**Section 2: Consultations for eczema – what's important**

- What makes a ‘good’ / ‘successful’ consultation?

*(prompts: rapport, communication, feeling listened to, feeling understood, problems addressed)*

- What’s important to you when having a consultation about your eczema?
- What would make you feel unhappy or dissatisfied during or after a consultation?

**Section 3: Experiences and perceptions of teleconsultations for eczema**

**Experiences:**

- Can you tell me about your most recent teleconsultation and what it was like for you?

*(Allow patient to talk about the things that are most important to them, but if not*  *already covered ask about):*

- - *How did you feel about the care you received?*
  - *How was your child’s skin assessed during the*  *teleconsultation and did you feel this was done accurately?*
  - *What was it like communicating with the doctor over phone or video?*
  - *Did you need to seek additional help following your appointment e.g. GP or A&E?*
- How did this experience compare with other teleconsultations you have had?

*(Explore if they have experienced different types of teleconsultations and how they compared)*

- How did having teleconsultations for eczema compare with having face-to-face consultations?

**Views**

- What do you think are the advantages of a teleconsultation compared to F2F?
- What are the disadvantages of teleconsultations compared to F2F?
- What are the barriers to accessing teleconsultations compared to F2F?
- Do you think teleconsultations can be an effective way to manage children with eczema and why?
- Do you have any concerns about the safety of teleconsultations?

**Section 4: Preferences for different consultation types**

- Out of the different types of teleconsultation available (telephone, video or mobile app), which do you prefer? Why?
- If you had the choice what type of consultation would you choose for managing your/your child’s eczema? Why?

*(Prompt: what about a combination of teleconsultations and face-to-face?)*

- What factors that would influence this choice?

*(For example if you could be seen quicker by teleconsultation, severity of eczema)*

**Section 5: Acceptability of different consultation types**

- Do you think teleconsultations are an acceptable way to manage children and young people with eczema?
- What influences whether you find a type of appointment acceptable?
- How would you feel if your next dermatology appointment was a telephone consultation?
- How would you feel if your next dermatology appointment was a video consultation?
- How would you feel if your next dermatology appointment was a face-to-face consultation?

**Section 6: Optimising how we deliver teleconsultations**

- Earlier we discussed what is important to you in a consultation. How can this be achieved through a teleconsultation?
- What do you think should determine how a patient is seen for their next appointment?

*(Prompts: patient choice, how severe their eczema is, how long they have been under*  *follow-up, if they are on a systemic and well controlled)*

- What does an ideal teleconsultation for eczema look like to you?
- How could teleconsultations be improved?

**Section 7: Planning a trial to compare teleconsultations with face-to-face consultations**

We are planning a study that would compare teleconsultations with face-to-face consultations for children and young people with eczema under dermatology. This would involve a group of patients being followed up by teleconsultations and a group of patients being followed up by face-to-face appointments. We would measure how effective the two types of consultation are by measuring the patients’ eczema severity and how acceptable patients find them. To avoid the study being biased, patients would be randomly assigned to an appointment type – they wouldn’t get to choose whether they are followed up by teleconsultation or face-to-face appointment. We plan that the study would run for one year.

What are your initial thoughts about this study?

In your opinion, what type of teleconsultation should we include in the study?

How would you feel about being randomly assigned to an appointment type?

**Section 3: Conclusion**

- I think that’s everything I have to cover. Is there anything else you’d like to tell me or any final thoughts you’d like to follow up?
- We will be informing participants of the results from this study, is this something you would be interested to hear about?
- Thank you for participating. If you have any further questions about the interview please let us know.
