# Supplementary figures and images for: Teleconsultations for Eczema in CHildren (TECH) feasibility study: a mixed-methods study with adolescents and parents
Source: Skin Health Dis. 2026 Jan 30;6(2):125–37. doi: 10.1093/skinhd/vzaf123 (PMC13036726; doi:10.1093/skinhd/vzaf123)

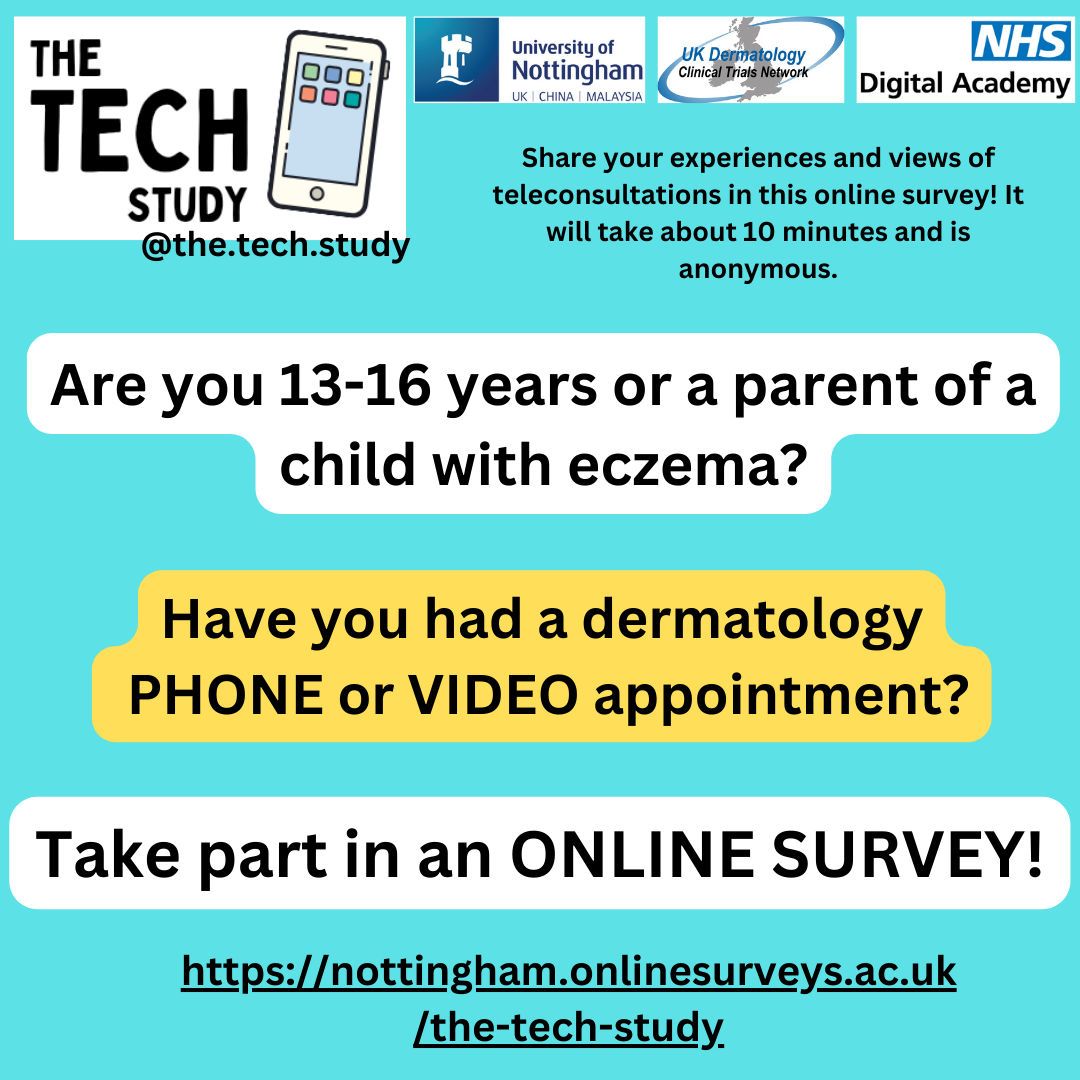

Supplement: vzaf123_Supplementary_Data [file vzaf123_supplementary_data.zip › Figure S1.png]
